# Gelsolin knockdown confers radiosensitivity to glioblastoma cells

**DOI:** 10.1002/cam4.7286

**Published:** 2024-05-27

**Authors:** Dezhi Gao, Tao Jiang, Yanwei Liu

**Affiliations:** ^1^ Beijing Neurosurgical Institute Capital Medical University Beijing China; ^2^ Department of Gamma‐Knife Center, Beijing Tiantan Hospital Capital Medical University Beijing China; ^3^ Department of Neurosurgery, Beijing Neurosurgical Institute Capital Medical University Beijing China; ^4^ Department of Neurosurgery, Beijing Tiantan Hospital Capital Medical University Beijing China; ^5^ Department of Radiation Oncology, Beijing Tiantan Hospital Capital Medical University Beijing China

**Keywords:** gelsolin, glioblastoma, radiosensitive, radiotherapy

## Abstract

**Objective:**

Radiotherapy (RT) is a cornerstone of the glioblastoma (GBM) treatment. However, the resistance of tumour cells to radiation results in early recurrence. The mechanisms underlying GBM radioresistance remain unclear. Screening for differentially expressed genes (DEGs) related to radiation might be a potential solution to this problem.

**Method:**

RT‐associated DEGs were screened based on the RNA sequencing of 15 paired primary and recurrent GBMs. The mRNA and protein expression of candidate genes were validated in RNA sequencing of The Chinese Genome Atlas (CGGA) dataset and 18 cases of GBM samples. The relationship between the candidate gene and radiation was confirmed in irradiated GBM cells. The association of candidate gene with clinical characteristics and survival was investigated in the CGGA and TCGA dataset. Biological function and pathway analysis were explored by gene ontology analysis. The association of the candidate gene with radiosensitivity was verified using cell counting Kit‐8, comet, and colony formation assays in vitro and subcutaneous tumour xenograft experiments in vivo.

**Results:**

Gelsolin (GSN) was selected for further study. GSN expression was significant elevated in recurrent GBM and up‐regulated in irradiated GBM cell lines. High expression of GSN was enriched in malignant phenotype of glioma. Moreover, high expression of GSN was associated with poor prognosis. Further investigation demonstrated that GSN‐knockdown (GSN‐KD) combined with RT significantly inhibited cell proliferation and enhanced radiosensitivity in vivo and in vitro. Mechanistically, GSN‐KD could lead to more serious DNA damage and promotes apoptosis after RT.

**Conclusion:**

Radiation induced up‐regulated of GSN. GSN‐KD could enhance the radiosensitivity of GBM.

## 1 INTRODUCTION

Glioblastoma (GBM) is the most prevalent and fatal tumour of the central nervous system. Radiotherapy (RT) is a cornerstone of GBM treatment.^1^, ^2^ Numerous randomised controlled trials have established level 1 evidence that RT improves patient survival. Despite appropriate RT, up to 90% of patients with GBM experience local relapse in a short time, indicating that the tumour is resistant to RT. Currently, the prognosis for GBM remains poor. The median survival time has been recorded to be only 14.6 months.^3^, ^4^, ^5^ Exploring potential impact mechanisms and sensitised targets is crucial for improving radiation therapy outcomes.

The fast development of bioinformatics and the rapid accumulation of gene expression datasets have provided significant research foundation for cancer research. Data analysis has been applied to several aspects of cancer research.^6^, ^7^ Identifying differentially expressed genes (DEGs) could provide reliable and specific biomarkers related with radiosensitivity. In head and neck squamous cell carcinoma Zhang, et al. identified four genes that associated with radioresistance.^8^ Jiang screened STC2 as a potential prognosis predictor of oesophageal squamous cell carcinoma and further confirmed that STC2 leads to radioresistance by promoting DNA damage repair (DDR) and inhibited ferroptosis.^9^ Yu et al. identified E2 F8 as transcription factor that contribute to GBM tumorigenesis, its regulatory mechanism to the CHEK1 and the oncogenic role was explored via bioinformatics analysis.^10^

In the present study, we found GSN was elevated in recurrent GBM and up‐regulated in irradiated GBM cell lines. High GSN expression was associated with malignant phenotype and shorter overall survival (OS). GSN‐knockdown (GSN‐KD) enhanced the radiosensitivity of GBM. This discovery is promising and provides a potential target for RT of GBM.

## 2 MATERIALS AND METHODS

### 2.1 Data and sample

In this article, three datasets were used for the analysis. The 15 paired RNA sequencing of primary and recurrent GBM samples (Table S1) was obtained from The Chinese Genome Atlas (CGGA). As reported in our previous study, two additional publicly available RNA sequencing datasets were downloaded from the CGGA database and TCGA data for validation of GSN expression and clinical characterisation, respectively.^11^

Furthermore, 18 cases of GBM samples were used to verify the protein expression and prognostic value of GSN. All patients with recurrent tumours had underwent RT. GBM tissues were obtained from patients who underwent neurosurgical resection at the Department of Neurosurgery at Tiantan Hospital. The tissues were frozen at −80°C before use and immersed in 4% paraformaldehyde to obtain paraffin‐embedded sections.

### 2.2 Cell culture

Three human GBM cell lines U87, U251 and LN229 were purchased from the Chinese Academy of Sciences Cell Bank and used for cytological experimenting in vitro. The cells were cultured in Dulbecco's modified Eagle's medium (DMEM) supplemented with 10% fetal bovine serum (FBS). Humidified incubators were supplied with atmosphere containing 5% carbon dioxide at 37°C.

### 2.3 X‐ray irradiation

GBM cells were irradiated with a Precision X‐ray Irradiator (PXi, USA) at a dose rate of 2.0 Gy/min at room temperature. Culture dishes or tumour‐loaded nude mice were placed under a collimator at a source‐to‐surface distance (SSD) of 50 cm, ensuring that the field size covered the culture dish or tumour.

### 2.4 Immunohistochemistry and evaluation of staining

The clinically acquired GBM samples were fixed in 4% formalin and embedded in paraffin. Paraffin‐embedded tissue blocks were sectioned (5 μm thick) onto slides and then de paraffinised. The primary antibodies against Gelsolin(1:150, ab109014)was applied overnight at 4°C. The positive cells and total number of cells per field were counted to determine the positive cell ratio.

### 2.5 Quantitative reverse transcription‐polymerase chain reaction (qRT‐PCR)

Total RNA was extracted using the RNeasy Mini Kit (Qiagen) according to the manufacturer's instructions. The RNA intensity was assessed using a 2100 Bioanalyzer (Agilent Technologies). The expression levels of the target genes were analysed using an ABI 7500 Real‐time PCR System. Transcript levels of GAPDH were normalised. The relative mRNA expression levels of the target genes were calculated using the comparative CT method, and the following GSN primer sequences were used:

5'‐GGTGTGGCATCAGGATTCAAG‐3' (sense) and 5'‐TTTCATACCGATTGCTGTTGGA‐3' (antisense).

The glyceraldehyde‐3‐phosphate dehydrogenase (GAPDH) was amplified with the following primers:

5'‐UGACCUCAACUACAUGGUUTT‐3' (sense) and 5'‐AACCAUGUAGUUGAGGUCATT‐3' (antisense).

### 2.6 Western blot analysis

Tumour tissues of subcutaneous tumour xenograft or cells were lysed on ice and total protein was extracted using RIPA lysis buffer (Beyotime Institute of Biotechnology). The protein concentration was measured using Coomassie Brilliant Blue (APPLYGEN A1011). Equal amounts of protein lysates (30 μg/lane) was subjected to electrophoresis on 10% SDS‐polyacrylamide gels and then transferred to PVDF membrane (cat. no. IPVH00010; EMD Millipore). The membranes were blocked with 5% milk (BD Biosciences) for 1 h and then incubated with corresponding primary antibody overnight at 4°C.The results were analysed using an enhanced chemi‐luminescence western blot detection system (Bio‐Rad Laboratories). In this study, the antibodies used were as followed: Rabbit anti‐gelsolin polyclonal antibody (1:1000; ab109014; abcam) Mouse anti‐γH2AX monoclonal antibody (1:1000; ab26350; abcam) Rabbit anti‐Caspase‐3 Monoclonal antibody (1:1000; ab184787; abcam).

### 2.7 Bioinformatics analysis

Gene ontology (GO) was used to analyse biological functions. Pearson's correlation analysis was used to examine the correlation between GSN mRNA expression and other genes using the R programming language. The positive correlated genes (*r* > 0.4, *p* < 0.05) were chosen for analysis using DAVID (http://david.abcc.ncifcrf.gov/home.jsp) to detect the biological processes that were correlated with GSN expression. The results are presented as a heatmap using the R programming language.

### 2.8 Establishment of cell line with GSN knockdown

We designed three GSN‐KD siRNA sequences (Table S2) and verified their knockdown effect by measuring protein expression. The most effective sequence was selected by the Gemma Company to construct lentiviral shRNAs. Sh‐U87 and a negative control were transfected into cells (multiplicity of infection [MOI] = 20) using 5 mg/mL polybrene transfection reagent (GenePharma, China). A concentration of 2 mg/mL puromycin was used to select stably transfected shU87 and NC cells. The cells were cultured until they were collected at a specific time for further studies.

### 2.9 Cell proliferation assay

To investigate the proliferation and sensitivity of Sh‐U87 and NC cells to radiation, cells that received different ionising radiation doses (0, 2, 4, 6, 8 and 10 Gy) were seeded in a 96‐well plate at a density of 1000 cells/well. A culture system was created by mixing 10 μL of CCK‐8 reagent with 90 μL of DMEM media. Then, each well received 100 μL of this system. CCK‐8 reagent was added according to the instructions of the CCK‐8 kit (Beyotime, China) and the cells were cultured for 2 h. The absorbance of cells was measured at 450 nm. This experiment was performed at 0, 2, 4, 8 and 10 days after RT.

### 2.10 Colony formation assay

Sh‐U87 and NC cells were grown for 24 h. Then the cells were plated into a 6‐well plate (1 × 10e3 cells per well). The cells received different ionising radiation doses (0, 2, 4, 8Gy) and were cultured for 14 days at 37°C in an incubator with 5% CO2. The colonies obtained were washed with PBS, fixed in 10% formalin for 10 min at room temperature, stained with Giemsa stain, and counted (50 cells). The results were analysed using GraphPad Prism version 5.0 and biological parameters of the radiation and survival curves were obtained.

### 2.11 Comet assay

DNA damage was assessed using the neutral comet assay.^12^ Sh‐U87 and NC cells were cultured in 3 cm^2^ culture dish for 24 h. Equal numbers of cancer cells were seeded in six‐well culture plates and irradiated with or without 6 Gy X‐rays of irradiation. In brief, after irradiation, portions of the cells were combined with low‐melting‐point agarose and transferred onto glass slides pre‐coated with high‐melting‐point agarose. Gel electrophoresis was performed 4 h after irradiation. 80–100 nuclei were counted for each slide. After electrophoresis, staining was carried out with the DAPI incubated at room temperature for 15 mins. The tail length was analysed by CASP software.

### 2.12 In vivo experiment

Animal experiments were performed in the animal laboratory of the Beijing Neurosurgical Institute according to the NIH guidelines. Twelve female BALB/c nude mice (6 weeks old) were obtained from Vital River Laboratory Animal Technology Co. Ltd (Beijing, China). Sh‐U87 or NC cells (1 × 10e6) were injected under the skin of the back flank to construct the tumourigenesis model, with six mice in each group. Tumour dimensions were measured using Vernier callipers every 2 days and calculated as (tumour width × tumour length)/^2^ RT was performed three weeks after subcutaneous tumour xenograft, the RT was carried out with Precision X‐ray Irradiator (PXi, USA) at a dose rate of 2.0 Gy/min. Nude mice were irradiated with a dose of 14Gy (in 2 Gyx7 fractions). The mice were euthanised 15 days after RT. The subcutaneous tumours were excised and analysed.

## 3 STATISTICAL ANALYSIS

All statistical analyses were conducted using the SPSS 16.0, R programming language 3.2.5, and GraphPad Prism 7.0 software. Student's *t* test was used to compare the expression of GSN in patients with primary and recurrent GBM or differential expression after RT. The prognostic significance was assessed by Kaplan–Meier survival analysis, with *p* < 0.05 (two‐side) considered statistically significant.

## 4 RESULTS

### 4.1 GSN expression was elevated in recurrent glioblastoma

**FIGURE 1:**
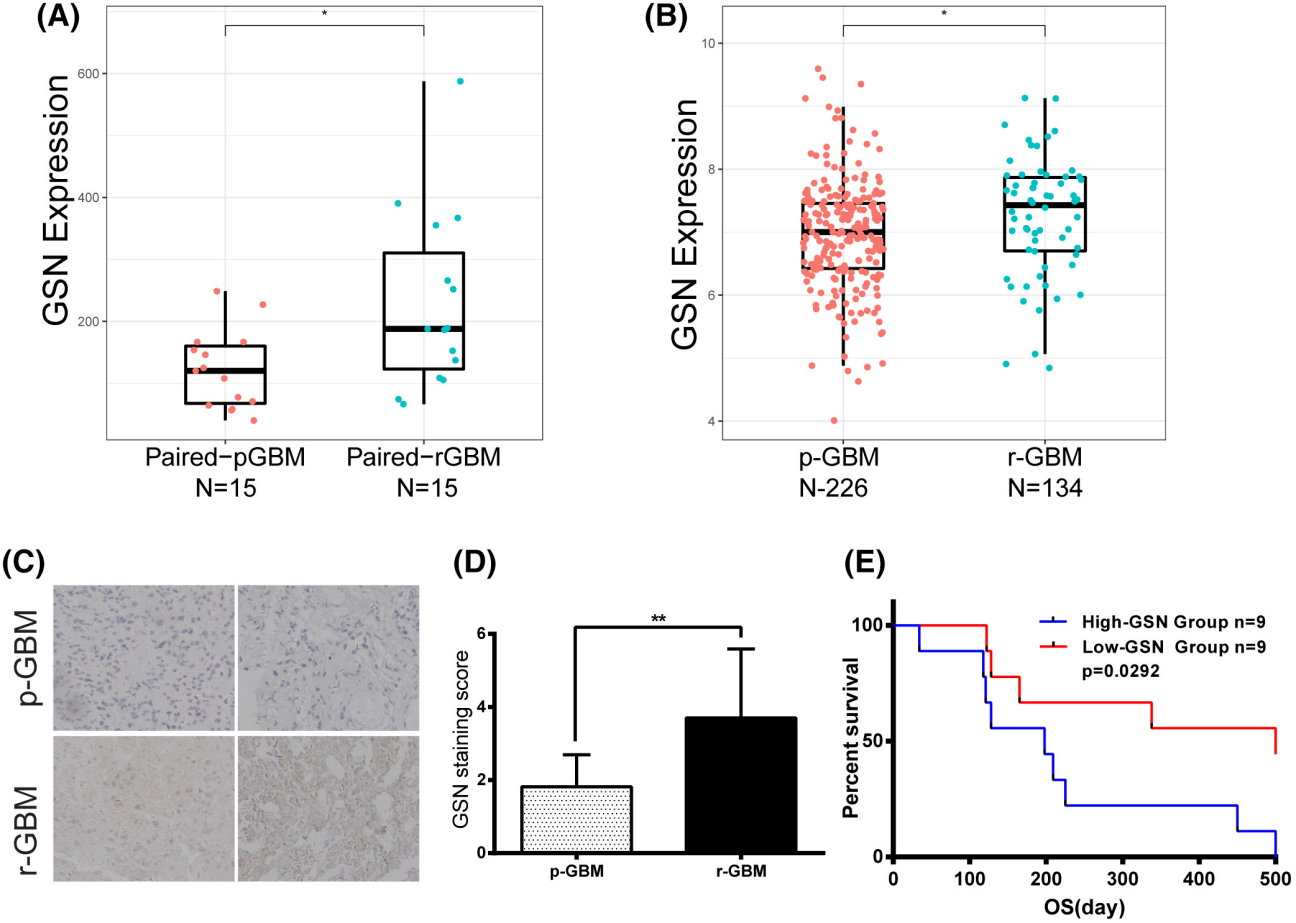
Gelsolin (GSN) expression was elevated in recurrent glioblastoma (GBM). (A) The expression levels of GSN were analysed in 15 paired pGBM and rGBM of the Chinese Genome Atlas (CGGA) mRNA sequencing datasets. (B) The up‐regulation of GSN in recurrent GBM was verified in 226 primary and 134 recurrent samples of the CGGA mRNA sequencing datasets. (C, D) The immunohistochemistry of GSN expression in 18 cases of GBM samples (Mag: 400×). (E) Kaplan–Meier curves of 18 cases GBM patient survival based on the expression level of GSN in CGGA. pGBM, primary GBM; rGBM, recurrent GBM. *p < 0.05; **p < 0.01.

The results revealed that four genes (Gelsolin, TMEM59L, ZBTB7A and ATX) were simultaneously up‐regulated in recurrent GBM.^11^ Among them, GSN showed the greatest difference and was therefore selected as the target gene in the present study (Figure 1A,B). To further verify GSN protein expression in GBM patients and its prognostic value, we performed IHC in 18 cases collected GBM tissue samples as well as Kaplan–Meier analysis in the corresponding patients. It was revealed that GSN protein levels was up‐regulated in the recurrent GBM that had underwent RT (Figure 1C,D). Moreover, the higher expression of GSN associated with shorter OS (Figure 1E).

### 4.2 Radiation induced increased GSN expression

**FIGURE 2:**
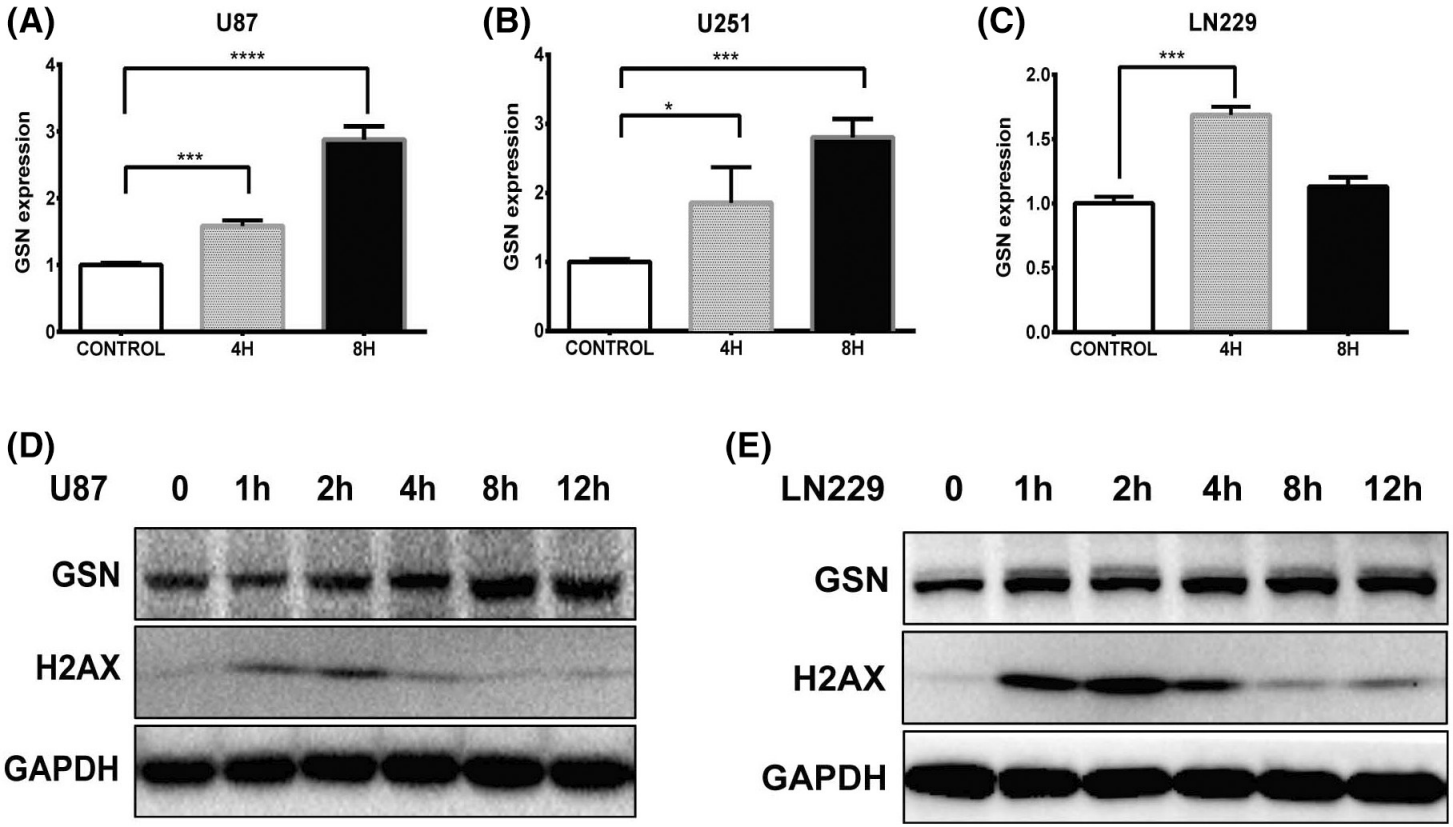
Radiation‐induced increasing expression of gelsolin (GSN). RNA expression of GSN was increased in (A) U87, (B) U251 and (C) LN229 cell lines after radiation. The protein expression of GSN was increased in (D) U87 and LN229 (E) cell lines after irradiation. *p < 0.05; ***p < 0.001; ****p < 0.0001.

To determine the relationship between GSN expression and RT. We measured GSN mRNA and protein expression of GSN in three irradiated GBM cell lines (U87, U251 and LN229). After a single irradiation with 5Gy, the expression of GSN was analysed by qPCR (at 0, 4 and 8 h) and western blotting (at 0, 1, 2, 4, 8 and 12 h). γ‐H2AX, as a marker of DSBs, was also evaluated. The result showed that the RNA expression of GSN was significantly up‐regulated in U87, U251 and LN229 cell line at 4 h after radiation. In the U87 and U251 cell lines, GSN showed a sustained elevated expression 8 h after irradiation (Figure 2A–C). The protein expression of GSN gradually increased from 4 to 8 h in U87 and LN229 cell lines after irradiation (Figure 2D,E). As a control, γ‐H2AX expression increased significantly within 1–4 h of irradiation (Figure 2D,E). This result indicates that radiation leads to the increased expression of GSN.

### 4.3 GSN expression in glioma and its clinicopathological features

Through RNA sequencing data analysis, we evaluated the relationship between GSN expression levels, *IDH* mutations, and MGMT methylation status in glioma obtained from the CGGA database. It was revealed that GSN was enriched in *IDH*‐wild‐type (Figure S1A,D) and MGMT unmethylated glioma(Figure S1B,E). We also analysed GSN expression in different molecular subtypes. The mesenchymal subtypes showed the highest GSN expression than the other three subtypes (Figure S1C,F). According to previous studies, *IDH*‐wild‐type, MGMT‐unmethylated and mesenchymal subtypes glioma generally exhibit poor prognosis. This finding indicates that the over‐expression of GSN was associated with the malignant phenotype.

### 4.4 High GSN expression associated with poor prognosis

**FIGURE 3:**
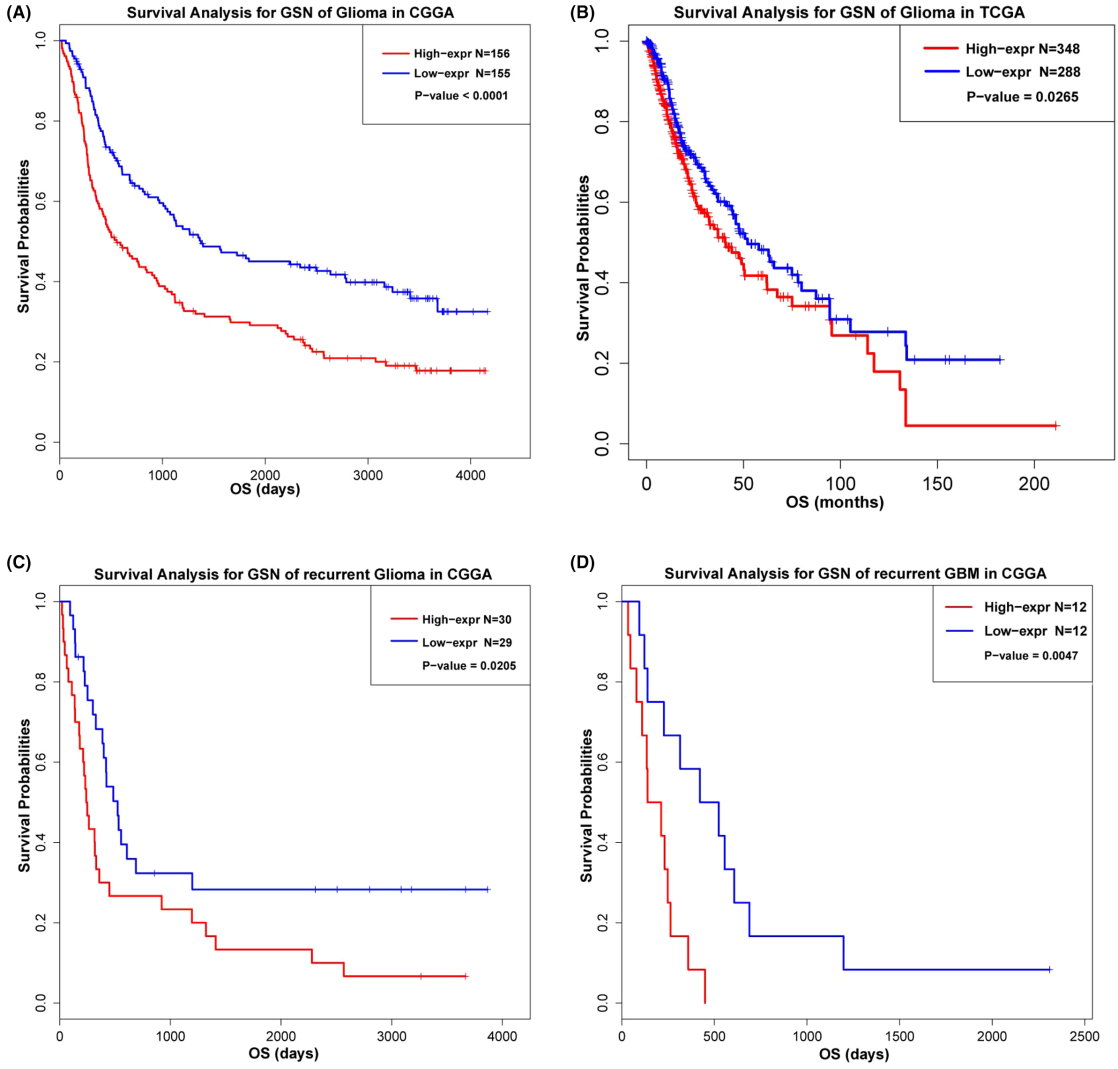
High gelsolin (GSN) expression associated with poor prognosis. (A, B) Survival analysis in glioma from the Chinese Genome Atlas (CGGA) and TCGA databases. (C, D) Survival analysis in recurrent glioma and recurrent glioblastoma (GBM) from the CGGA databases.

To evaluate the prognostic value of GSN expression in glioma patients, Kaplan–Meier survival analysis was performed with the data from the CGGA and TCGA RNA sequencing datasets. Our data revealed that overexpression of the GSN appears significantly correlated with unfavourable OS in glioma. Similar results was observed in the TCGA dataset (Figure 3A,B). Considering RT leads to increase expressed of GSN in recurrent GBM, we additionally analysed the prognostic value of GSN in recurrent glioma and GBM patients in CGGA dataset. Consistent with the result in glioma, the higher GSN expression exhibited a significantly shorter survival than lower GSN expression in recurrent glioma and GBM (Figure 3C,D). These results revealed that GSN is a negative prognostic factor in glioma and GBM patients.

### 4.5 GSN related biological process

**FIGURE 4:**
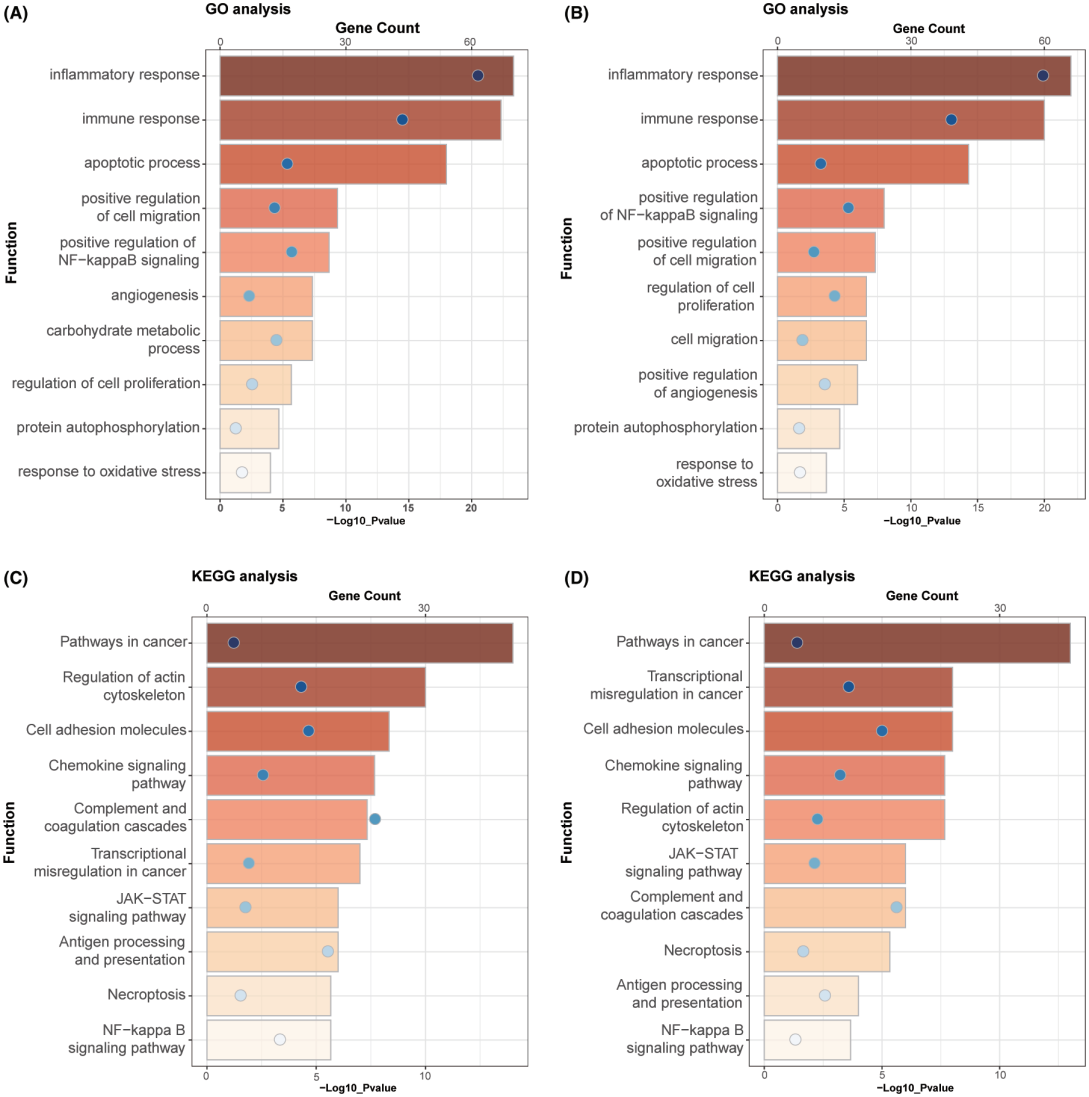
Biological function and pathway analysis of gelsolin (GSN) in The Chinese Genome Atlas (CGGA) and TCGA datasets. (A, B) Gene ontology analysis of GSN expression in glioma. Genes that positively correlated with GSN expression were highly enriched in the inflammatory response, immune response, apoptotic process, regulation of cell proliferation and migration, regulation of NF‐κB signalling, angiogenesis and response to oxidative stress in GO terms. (C, D) KEGG analysis of GSN expression in glioma. The samples were ranked according to count number. The bar charts represented the count and the circle represented the *p*‐value. (Pearson |R| >0.4; *p* < 0.05).

Next, we further investigated the biological process that associated with GSN expression. Pearson correlation analysis was performed to identify the genes that tightly correlated with GSN expression (Pearson |R| >0.4) in the CGGA and TCGA sequencing datasets. Significantly related genes were used for gene ontology (GO) analysis with DAVID. According to the results shown in Figure 4A,B, genes that positively correlated with GSN expression were highly enriched in the inflammatory response, immune response, apoptotic process, regulation of cell proliferation and migration, regulation of NF‐κB signalling, angiogenesis and response to oxidative stress in GO terms. The KEGG pathway analysis revealed that GSN expression is positively related to the pathways in cancer, cell adhesion molecules, chemokine signalling pathway antigen processing and presentation, necroptosis, JAK−STAT signalling pathway and NF‐κB signalling pathway (Figure 4C,D). All the results mentioned above were shared by the two datasets. These analyses suggesting that GSN might functionally important for cancer progression, immune response and apoptosis process.

### 4.6 GSN knockdown confers radiosensitivity of GBM

**FIGURE 5:**
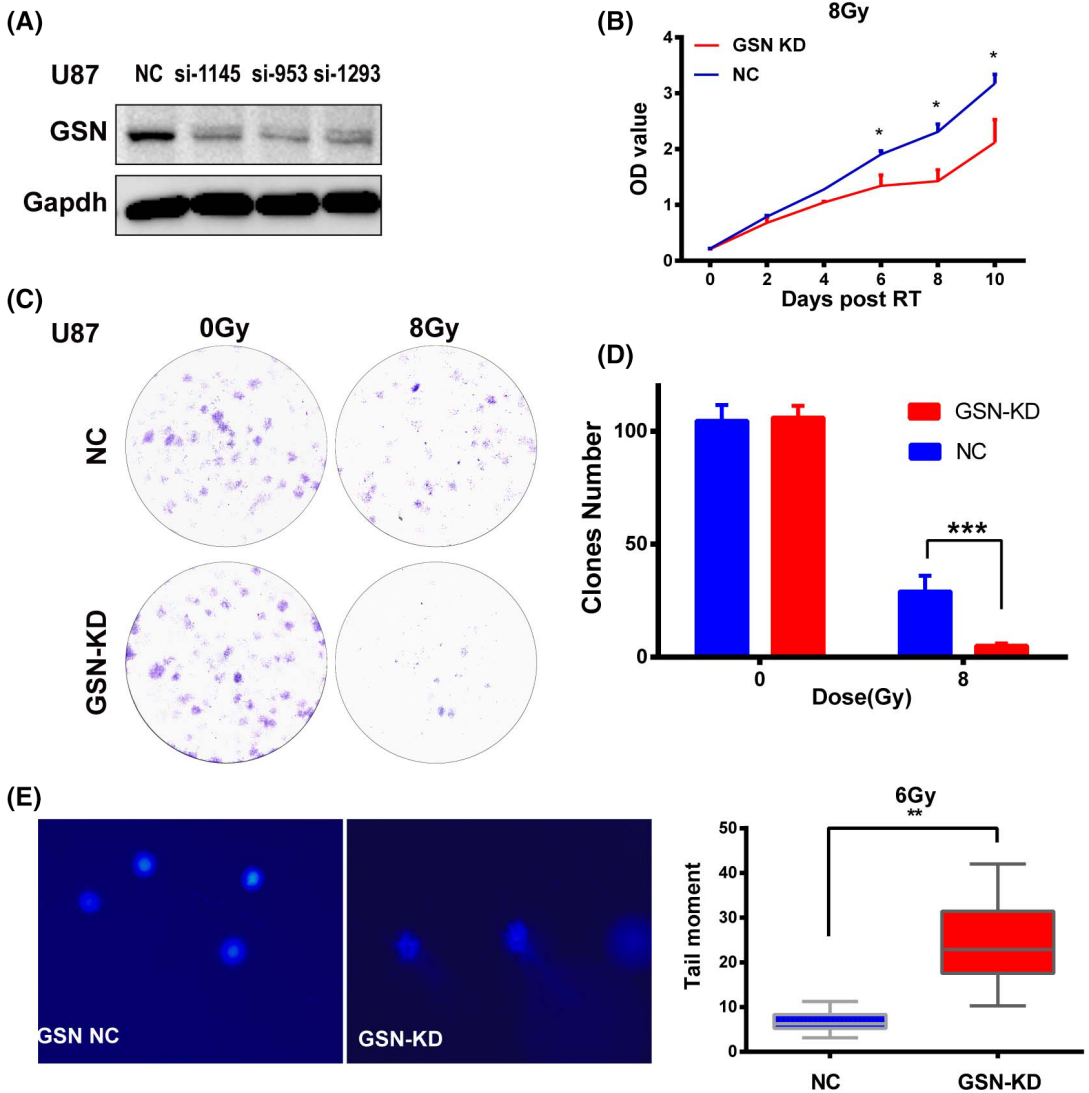
Gelsolin‐knockdown (GSN‐KD) confers radiosensitivity to glioblastoma cells. (A) The protein level of GSN was detected in GSN‐KD and NC cells. (B) Cell viability after radiation was determined in GSN‐KD and NC group by CCK8 assay. (C, D) Colony formation assays of GSN‐KD and NC cells after irradiation with 0 and 8 Gy. The cell colony count in each group. (E) Comet assay was carried out in GSN‐KD and NC cells at 4 h after 6Gy RT. *p <0.05, **p <0.01, ***p <0.001.

**FIGURE 6:**
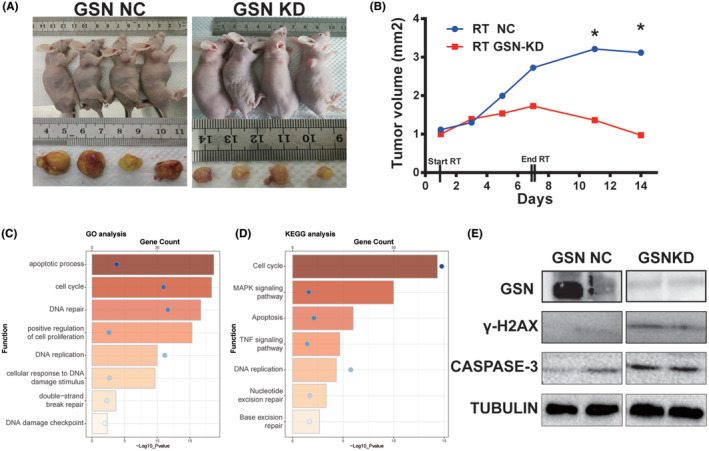
Gelsolin‐knockdown (GSN‐KD) enhanced radiosensitivity in glioblastoma (GBM) in vivo. (A) General view of tumour mass in each indicated group at the time point of the last radiotherapy (mice) and end point of the experiment (tumour). (B) Size change curves of xenograft tumours in each group after RT. (C, D) Biological function and pathway analysis of GSN‐KD and NC group in the xenograft tumours. (E) The protein expression of γ‐H2AX and casepase‐3 was increased in GSN KD group after RT. *p < 0.05.

We then established stable GSN‐KD and empty vector transfected U87 cell line to performed experiment in vitro and in vivo so as to observe the GSN‐affected radiosensitive in GBM cell. The knockdown efficacy was verified by western blotting (Figure 5A). Using stable GSN‐KD and NC U87 cell, CCK‐8, colony formation and comet assay were performed in vitro. It was demonstrated cell viability was significantly reduced (Figure 5B) and diminished colony formation after irradiated with 8Gy (Figure 5C,D). The comet assay results showed that the olive tail moment is higher in GSN‐KD cell than in NC group, which indicates that radiation induced more severe DNA damage (Figure 5E). In vivo experiment, 3 weeks after injected with GSN‐KD and U87 NC cells, xenograft tumour models were successful prepared in 12 mice and RT was administered. Nude mice were irradiated with a dose of 14 Gy (2 Gy ×7 fractions). The final tumour size data were collected on the 14th day after RT and subcutaneous tumours were extracted. The size change curves showed a discrepancy between the GSN‐KD and NC groups. The tumour size in the GSN‐KD group was smaller than that in the NC group, suggesting that GSN‐KD strengthened the effect of radiation on tumour suppression in vivo (Figure 6A,B).

### 4.7 GSN‐KD increased DNA damage and promoted apoptosis

Next, we performed RNA‐sequencing analysis using the subcutaneous tumour tissue. We found that the lower expressed process in GSN‐KD group were highly enriched in the apoptotic process, cell cycle, DNA repair and replication, DNA damage checkpoint in GO terms. The KEGG pathway analysis revealed that the pathway with reduced expression were closely related to the cell cycle, MAPK signalling pathway, apoptosis, TNF signalling pathway, DNA replication, nucleotide and excision repair in the GSN‐KD group (Figure 6C,D). As is well known, DNA damage and apoptosis are closely related to radiosensitivity.^13^, ^14^, ^15^, ^16^ Then we focused on the DNA damage and apoptosis‐related DEGs annotation and particular pathway so as to preliminary seek for potential mechanisms affecting radiosensitivity. The protein expressions of GSN, casepase‐3 and γ‐H2AX were measured in the xenograft tumours after RT. The results showed the expression of γ‐H2AX and casepase‐3 was increased in GSN‐KD group (Figure 6E). Combining the above results, it suggested that GSN‐KD coordinate with RT leads to more serious DNA damage and promotes apoptotic process.

## 5 DISCUSSION

The mechanism of radioresistance in GBM have been identified regulated by various cellular signalling mechanisms, including DDR, cell cycle regulation,^7^, ^8^ and cell apoptosis.^9^, ^10^ However, effective radiosensitizer that can increase the efficacy of RT have not yet been developed. In the current research, we identified a RT related DEG—GSN by combining examination of RNA sequencing and bioinformatics analysis of GBM. We further demonstrated GSN‐KD confers radiosensitivity of GBM. This discovery is promising and provides a potential target for RT of GBM.

GSN is an actin‐binding protein that can regulate the length of actin filaments.^17^, ^18^ A previous study reported the prognostic value of GSN in various tumours. GSN expression is down‐regulated in 60%–90% of tumours during carcinogenesis in the breast, colon, stomach, bladder, prostate and lungs. However, many results generated to date are controversial regarding the role of GSN in different carcinomas.^19^, ^20^ Regarding glioma, Wang et al reported GSN expression was significant elevated in lower grade glioma and GBM contrasted with in healthy tissue.^21^ On the contrary, Zhang el al reported lower expression of GSN in GBM tissues than the healthy brain tissues.^22^ In the current study, we found GSN was elevated in recurrent GBM and up‐regulated in irradiated GBM cell lines. In addition, our results indicate that overexpression of GSN was associated with malignant phenotype of GBM. Similar result was reported in the study of hepatocellular carcinoma patients.^23^ These results indicate that GSN expression was associated with the development and malignancy progression of glioma. Moreover, several reports suggest that GSN may be related to radiosensitivity. Kim et al identified gelsolin was up‐regulated protein after radiation‐exposed and suggesting it may play radioresistant and negative roles in cancer therapy.^24^ GSN up‐regulation is associated with radio‐or chemoresistance in non‐small cell lung cancer and gynaecological cancer cells.^18^, ^25^ In the present study, we demonstrated radiation induced increased GSN expression. The results of survival analysis show that the high GSN expression associated with poorer prognosis in glioma and GBM patients (Figures 1E and 3A–D). We speculated that the expression of GSN might related to radiosensitivity in GBM. Until now, there has been no study concerning the role of GSN in radiosensitivity of GBM.

Next, we performed a series of bioinformatics analysis. As shown in Figure 4, GSN was tightly associated with the immune and inflammatory response, cell proliferation and cell migration, which are key steps for tumour progression. Meanwhile, the KEGG analysis demonstrated that GSN was positively related to the antigen processing and presentation, JAK–STAT signalling pathway and NF‐κB signalling pathway. This indicates that GSN may participate in the pathway of promoting cell survival, its silencing could be used to block this signalling pathway.

Moreover, our study demonstrated that GSN‐KD confers radiosensitivity to GBM cells. GSN‐KD remarkably attenuated cell viability (Figure 5B) and diminished colony formation after RT in vitro (Figure 5C,D). The tumour size in the GSN‐KD group was smaller than that in the NC group after RT in vivo (Figure 6A,B). Then, using tumour tissue, we performed a series of bioinformatics analysis. The result revealed GSN‐KD was closely correlated with the cell cycle, DNA repair and apoptosis (Figure 6C,D), which played important role in radiosensitivity. The Western blot results of the mice tumour tissue showed that the expression of γ‐H2AX increased in the GSN‐KD group (Figure 6E). Considering the results of the comet experiment, these results indicate that GSN‐KD leads more severe DNA damage response to RT.

In addition, as a prominent caspase‐3 substrate, GSN is closely associated with apoptosis in vitro.^17^, ^26^ However, the inhibiting or promoting effect of GSN may vary depends upon the type of tissues and cells.^21^, ^27^, ^28^ The Western blot results showed increasing expressed of casepase‐3 in the GSN‐KD group, indicating activation of the apoptotic pathway. These results indicate that revealing the mechanism of GSN‐KD confers radiosensitivity to GBM cells might achieve by affecting DNA damage and regulating apoptosis process.

Although we observed an effect of GSN expression on the radiosensitivity of GBM, the specific mechanism remains unclear. U87MG cells harbour a mutationally inactivated PTEN gene, which is the primary negative regulator of the PI3K‐AKT signalling pathway. Further experiments are required to determine the genetic status of PTEN and other factors critical to this signalling pathway. Future research will focus on in‐depth studies of the signalling pathways and interacting genes.

## 6 CONCLUSION

In conclusion, radiation induced up‐regulation of GSN in GBM. GSN‐KD could enhance the radiosensitivity of GBM. Our research suggests that GSN might be an attractive therapeutic target for overcoming GBM radioresistance.

## AUTHOR CONTRIBUTIONS

**Dezhi Gao:** Conceptualization (equal); data curation (equal); formal analysis (equal); investigation (equal); methodology (equal); project administration (equal); resources (equal); software (equal); validation (equal); visualization (equal); writing – original draft (equal); writing – review and editing (equal). **Tao Jiang:** Conceptualization (equal); data curation (equal); formal analysis (equal); funding acquisition (equal); investigation (equal); methodology (equal); project administration (equal); resources (equal); supervision (equal); validation (equal); writing – review and editing (equal). **Yanwei Liu:** Conceptualization (equal); data curation (equal); formal analysis (equal); funding acquisition (equal); investigation (equal); methodology (equal); project administration (equal); resources (equal); software (equal); supervision (equal); validation (equal); visualization (equal); writing – review and editing (equal).

## FUNDING INFORMATION

The authors disclosed receipt of the following financial support for the research, authorship, and/or publication of this article: This work was supported by the grants from The National Natural Science Foundation of China (Grant Numbers: 82172771). The funding sources had no influence on the design, performance, or reporting of this study.

## CONFLICT OF INTEREST STATEMENT

The authors declare that there are no conflict of interests.

## ETHICS STATEMENT

The studies involving animal experiment were reviewed and approved by Ethics Committee of the Beijing Institute of Neurosurgery. This study was approved by the Ethical Review Committee of Beijing Tiantan Hospital (IRB KY2013‐017‐01).

## CONSENT

Written informed consent was obtained from all the patients, for the publication of any potentially identifiable data included in this article.

## Supporting information


Figure S1.

Table S1.

Table S2.

## Data Availability

The datasets used and/or analysed during the current study is available online or available from the corresponding authors on reasonable request.
