# Supplementary figures and images for: Gelsolin knockdown confers radiosensitivity to glioblastoma cells
Source: Cancer Med. 2024 May 27;13(10):e7286. doi: 10.1002/cam4.7286 (PMC11130581; doi:10.1002/cam4.7286)

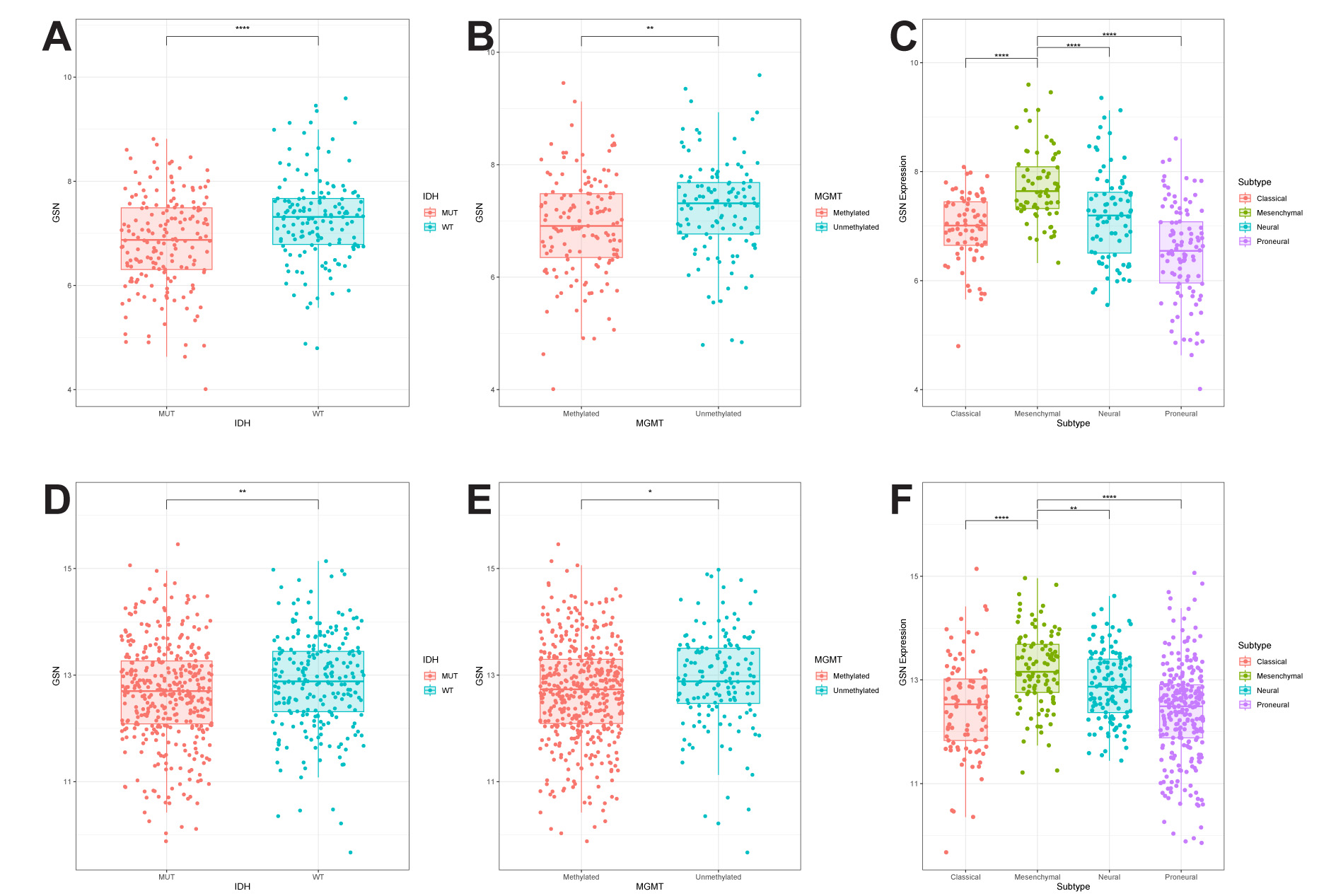

Supplement: Supplementary file 1 — Figure S1. [file CAM4-13-e7286-s003.jpg]
