# Supplementary material for: Gelsolin knockdown confers radiosensitivity to glioblastoma cells
Source: Cancer Med. 2024 May 27;13(10):e7286. doi: 10.1002/cam4.7286 (PMC11130581; doi:10.1002/cam4.7286)
Supplement: Supplementary file 2 — Table S1. [file CAM4-13-e7286-s002.docx]

**Supplemental Table-1**

15 pairs of paired data‘s ID number in the CGGA database.

| Pairing number | Data category | Sample ID  in CGGA | Source of data sets | Data category | Sample ID  in CGGA | Source of data sets |
| --- | --- | --- | --- | --- | --- | --- |
| PD4 | pGBM | CGGA_1024 | CGGA 325 data set | rGBM | CGGA_1337 | CGGA 693 data set |
| PD9 | pGBM | CGGA_1049 | CGGA 325 data set | rGBM | CGGA_1257 | CGGA 693 data set |
| PD12 | pGBM | CGGA_1103 | CGGA 693 data set | rGBM | CGGA_1381 | CGGA 325 data set |
| PD15 | pGBM | CGGA_1114 | CGGA 325 data set | rGBM | CGGA_1248 | CGGA 693 data set |
| PD16 | pGBM | CGGA_1124 | CGGA 325 data set | rGBM | CGGA_1255 | CGGA 693 data set |
| PD22 | pGBM | CGGA_1214 | CGGA 325 data set | rGBM | CGGA_1541 | CGGA 693 data set |
| PD28 | pGBM | CGGA_1287 | CGGA 325 data set | rGBM | CGGA_1505 | CGGA 693 data set |
| PD31 | pGBM | CGGA_1353 | CGGA 693 data set | rGBM | CGGA_1682 | CGGA 693 data set |
| PD35 | pGBM | CGGA_1403 | CGGA 693 data set | rGBM | CGGA_1638 | Unpublished dataset |
| PD36 | pGBM | CGGA_1410 | CGGA 693 data set | rGBM | CGGA_1702 | CGGA 693 data set |
| PD46 | pGBM | CGGA_1687 | CGGA 693 data set | rGBM | CGGA_1911 | CGGA 693 data set |
| PD48 | pGBM | CGGA_1749 | CGGA 693 data set | rGBM | CGGA_1815 | CGGA 693 data set |
| PD49 | pGBM | CGGA_1758 | CGGA 693 data set | rGBM | CGGA_1865 | CGGA 693 data set |
| PD72 | pGBM | CGGA_604 | CGGA 325 data set | rGBM | CGGA_777 | CGGA 693 data set |
| PD73 | pGBM | CGGA_680 | CGGA 325 data set | rGBM | CGGA_P12 | Unpublished dataset |
