# Supplementary material for: Gelsolin knockdown confers radiosensitivity to glioblastoma cells
Source: Cancer Med. 2024 May 27;13(10):e7286. doi: 10.1002/cam4.7286 (PMC11130581; doi:10.1002/cam4.7286)
Supplement: Supplementary file 3 — Table S2. [file CAM4-13-e7286-s001.doc]

Supplemental Table-2

The primer sequences for si-GSN were the following:

| Homo-1293 | Sense, 5′- GCU GUU GAG GUA UUG CCU ATT-3′ |
| --- | --- |
|  | Antisense, 5′- UAG GCA AUA CCU CAA CAG CTT -3′ |
| Homo-1145 | Sense, 5′-GCC UCU GAC UUC AUC ACC ATT-3′ |
|  | Antisense, 5′- UGG UGA UGA AGU CAG AGG CTT-3′ |
| Homo-953 | Sense, 5′-GCC AAG CUC UAC AAG GUC UTT-3′ |
|  | Antisense, 5′- AGA CCU UGU AGA GCU UGG CTT-3′ |
| NC | Sense, 5′- UUC UCC GAA CGU GUC ACG UTT-3′ |
|  | Antisense, 5′- ACG UGA CAC GUU CGG AGA ATT-3′ |
| GAPDH | Sense, 5′- UGA CCU CAA CUA CAU GGU UTT-3′ |
|  | Antisense, 5′- AAC CAU GUA GUU GAG GUC ATT-3′ |
